# Impact of the Peptide WMR-K on Dual-Species Biofilm *Candida albicans/Klebsiella pneumoniae* and on the Untargeted Metabolomic Profile

**DOI:** 10.3390/pathogens10020214

**Published:** 2021-02-16

**Authors:** Emilia Galdiero, Maria Michela Salvatore, Angela Maione, Federica Carraturo, Stefania Galdiero, Annarita Falanga, Anna Andolfi, Francesco Salvatore, Marco Guida

**Affiliations:** 1 Department of Biology, University of Naples ‘Federico II’, via Cinthia, 80126 Naples, Italy; egaldier@unina.it (E.G.); angela.maione@unina.it (A.M.); federica.carraturo@unina.it (F.C.); marco.guida@unina.it (M.G.); 2 Department of Chemical Sciences, University of Naples ‘Federico II’, via Cinthia, 80126 Naples, Italy; andolfi@unina.it (A.A.); frsalvat@unina.it (F.S.); 3 Department of Pharmacy, School of Medicine, University of Naples ‘Federico II’, Via Domenico Montesano 49, 80131 Naples, Italy; sgaldier@unina.it; 4 Department of Agricultural Science, University of Naples ‘Federico II’, Via dell’ Università 100, 80055 Naples, Italy; annarita.falanga@unina.it; 5 BAT Center—Interuniversity Center for Studies on Bioinspired Agro-Environmental Technology, University of Naples ‘Federico II’, 80055 Naples, Italy

**Keywords:** polymicrobial, fungi, bacteria, metabolomics, GC-MS, footprinting

## Abstract

In recent years, the scientific community has focused on the development of new antibiotics to address the difficulties linked to biofilm-forming microorganisms and drug-resistant infections. In this respect, synthetic antimicrobial peptides (AMPs) are particularly regarded for their therapeutic potential against a broad spectrum of pathogens. In this work, the antimicrobial and antibiofilm activities of the peptide WMR-K towards single and dual species cultures of *Candida albicans* and *Klebsiella pneumoniae* were investigated. We found minimum inhibitory concentration (MIC) values for WMR-K of 10 µM for *K. pneumoniae* and of 200 µM for *C. albicans*. Furthermore, sub-MIC concentrations of peptide showed an in vitro inhibition of biofilm formation of mono and polymicrobial systems and also a good biofilm eradication even if higher concentrations of it are needed. In order to provide additional evidence for the effect of the examined peptide, a study of changes in extracellular metabolites excreted and/or uptaken from the culture medium (metabolomic footprinting) in the poly-microbial association of *C. albicans* and *K. pneumoniae* in presence and absence of WMR-K was performed. Comparing to the untreated dual species biofilm culture, the metabolomic profile of the WMR-K treated culture appears significantly altered. The differentially expressed compounds are mainly related to the primary metabolic pathways, including amino acids, trehalose, pyruvic acid, glycerol and vitamin B6.

## 1. Introduction

Biofilms are responsible for approximatively 80% of microbial infections in humans, and they have also been recovered from all sorts of habitats and surfaces, from aquatic environments to implanted medical devices and artificial industrial structures, passing through plant and mammalian tissues [1,2,3,4]. Microorganisms organized in biofilms undergo epigenetic changes in their state with respect to the planktonic form consisting of alterations in cell morphology, communication between cells, expression of some genes, production of an extracellular matrix made of carbohydrates, proteins and nucleic acids and, above all, evidence of phenotypic characteristics, such as resistance to antimicrobial agents [5,6]. The latter is one of the most common features of microbial biofilms and, for this reason, diseases involving biofilms are generally chronic and difficult to treat with common antibiotics [7,8].

Biofilm phenotypic resistance is due to numerous mechanisms; some of them are crucial, such as drug sequestration by the biofilm matrix and up-regulation of drug efflux pumps, both resulting in lower intracellular concentrations of the antimicrobial drug [9,10].

Several microorganisms are capable of forming single-species biofilms, but it is much more frequent to find associations of two or more bacterial and/or fungal species. In fact, multiple microbial species are closely associated in the poly-microbial biofilms providing particular advantages to each species when compared with single-species biofilms [11,12]. 

The biofilm-mediated microbial association between the prokaryotic pathogen *Klebsiella pneumoniae* and the eukaryotic pathogen *Candida albicans* has particular medical relevance. Although the interaction between *C. albicans* and *K. pneumoniae* is quite uncommon, these pathogens have been shown to coexist within the human host as complex biofilm communities causing systemic infections and are one of the major causes of morbidity and mortality in hospitalized patients [13,14]. 

*C. albicans* is one of few fungal species acting as opportunistic pathogen and causing diseases in humans with various levels of severity ranging from superficial mucosal to dermal infections. These infections are linked to fungal capacity to adhere to and grow on biological and inanimate surfaces as biofilms [15]. In fact, this fungus produces highly structured biofilms containing metabolically inactive persister cells which increase the tolerance to fungicidal drugs [16]. The presence on biomaterials, such as stents, prostheses, catheters, pacemakers, of microorganisms that can form biofilms and some predisposing factors (e.g., advanced age, antibiotic or immunosuppressive therapies) are the basis for the onset of *C. albicans* [17]. 

*Klebsiella pneumoniae* is considered to be a major opportunistic pathogen for humans and one of the prominent cause of nosocomial infections due to its multi-drug resistance, which poses serious threat in the clinical practice [18]. It is involved in a variety of diseases, including pneumonia, urinary tract infection and wound infection. Its biofilm forming ability is the most important contributor to hospital-acquired and recurrent infections [19,20].

The increasing awareness of the importance to find novel antibiofilm and anti-virulence drugs to suppress dual biofilm formation and virulence has stimulated a huge research activity. Nowadays, it has become necessary also to look for alternative strategies that target only to microbial ability to form biofilms and to the production of virulence factors, without affecting planktonic cells growth [21,22].

Antimicrobial peptides (AMPs) represent an attractive alternative to conventional drugs for the development of new antimicrobials since they have shown to have broad-spectrum activity towards bacteria, fungi and viruses, furthermore, the resistance mechanisms towards them are limited [23].

AMPs although being highly diverse in sequence, length and structure present similar mechanisms of action, which involve the disruption of bacterial membrane integrity. Several models have been hypothesized for membrane disruption as reported by Kang et al. [24]; moreover, some AMPs are able to cross the bilayer and target important functions of bacterial metabolism.

The antibacterial WMR peptide, which is a modification of the native sequence of myxinidin, a marine peptide isolated from the epidermal mucus of hagfish (*Myxine glutinosa* L.), showed a potent antimicrobial activity against a wide range of bacteria and yeast, and it demonstrated high levels of activity against *Pseudomonas aeruginosa* and low levels of cytotoxicity against human cells [25,26,27]. A later modification of the WMR sequence, the analogue WMR-K, was developed to enable peptide covalent binding to self-assembled peptide fibers for intracellular delivery. The obtained nanofiber structures significantly inhibit biofilm formation and eradicate the already formed biofilms *of P. aeruginosa* (Gram-negative bacteria) and *C. albicans*. Our results provided insights into the design of peptide based supramolecular assemblies with antibacterial activity and established an innovative strategy to develop self-assembled antimicrobial materials for biomedical applications. The peptide WMR-K is thus an interesting molecule to be further developed for its antimicrobial and antibiofilm activities [28]. 

The aim of this work is to investigate the antimicrobial and antibiofilm activities of the peptide WMR-K towards single and dual species cultures of *K. pneumoniae* and *C. albicans*. Further investigations on the effect of this modified peptide on the dual species biofilms were performed by GC-MS-based metabolomics analysis. In fact, microorganisms produce and/or excrete several metabolites with different chemical structures and biological functions belonging to primary and secondary metabolic pathways [29,30,31]. These metabolites reflect the physiological state of the microorganisms in response to biotic and abiotic stimuli [32,33]. In this respect, the untargeted analysis of metabolite uptake and excretion from the culture medium, also known as metabolomic “footprinting” [34,35,36], is particularly relevant to study the whole group of metabolites which respond to the presence of active substances (e.g., AMPs) [37]. 

## 2. Results

### 2.1. Antimicrobial and Antibiofilm Activities

**Figure 1:**
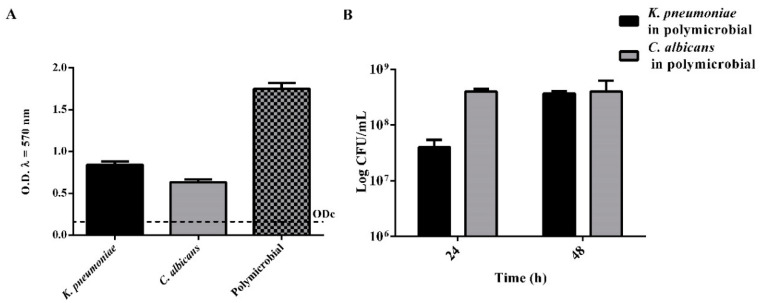
Biofilm characterization. (**A**) Biofilm growth of single and dual species microorganisms. (*n* = 3, ± SD). OD_cut_ = mean of negative control with 3 times addition of SD. (**B**) Quantification of viable cell of *Klebsiella pneumoniae* and *Candida albicans* in polymicrobial biofilm at 24 and 48 h (*n* = 3, ± SD).

As showed in Figure 1, both *K. pneumoniae* and *C. albicans* are able to form in vitro biofilms based on the classification of adherence capabilities reported by Stepanovic et al. [38]. The dual-species biofilm, obtained by co-culturing both microorganisms, resulted in a strong degree of biofilm enhancement, which was estimated (CFU·mL^−1^) of 30% of *K. pneumoniae* and 70% of *C. albicans* at 24 h, while reached an equivalent composition, respectively, of 47 and 53% at 48 h (Figure 1B).

The effect of the peptide WMR-K was evaluated on planktonic cells and mono/polymicrobial biofilm cultures of *K. pneumoniae* and *C. albicans*.

**Figure 2:**
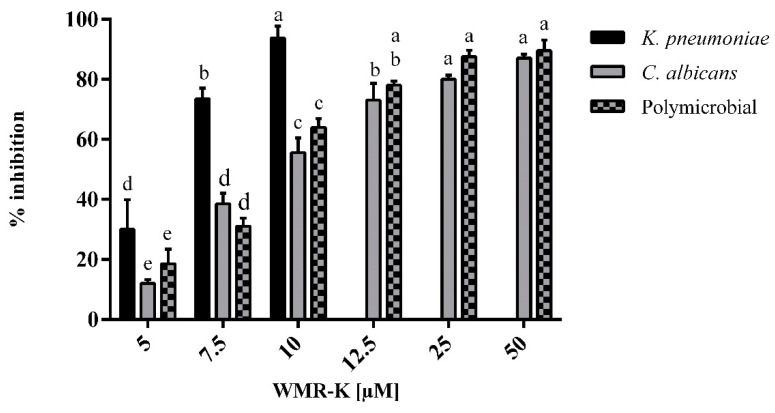
Inhibition effects of WMR-K at different concentrations on mono and poly-microbial biofilms of *K. pneumoniae, C. albicans* in 96-well microplate. Sub-MIC concentrations of WMR-K (5, 7.5, and 10 μM for *K. pneumoniae* biofilm; 7.5, 10, 12.5, 25, and 50 μM for *C. albicans* and for dual-species biofilms) were incubated with microorganisms as a preventive strategy; (*n* = 3 ± SD); data with different letters (**a**–**e**) are significantly different (two-way ANOVA followed by Tukey’s post hoc test, *p* < 0.05). Values with dissimilar letters are significantly different from each other (*p* < 0.05). Values with the same letter are not significantly different (*p* > 0.05).

The minimum inhibitory concentration (MIC) of the peptide against *C. albicans* was found to be 200 μM. However, WMR-K at a concentration of 50 μM slightly reduced the planktonic cell growth of *C. albicans* (by 1 log CFU·cm^−2^) (Supplementary Figure S1). In addition, WMR-K at concentrations of 12.5 and 25 μM inhibited *C. albicans* biofilm formation by 75 and 80%, respectively (Figure 2).

**Figure 3:**
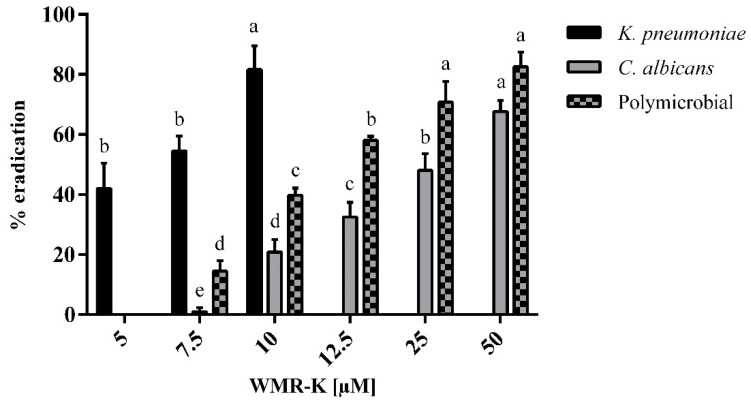
Reduction of biofilm metabolic activity of WMR-K at different concentrations on preformed mono and poly-microbial biofilms of *K. pneumoniae, C. albicans* in 96-well microplate. (*n* = 3 ± SD). Fresh growth medium containing sub-MIC concentrations of WMR-K (5, 7.5, and 10 μM for *K. pneumoniae* biofilm; 7.5, 10, 12.5, 25, and 50 μM for *C. albicans* and for dual-species biofilms) were added in the mature biofilm and incubated for another 24 h. Data with different letters (**a**–**e**) are significantly different (two-way ANOVA followed by Tukey’s post hoc test, *p* < 0.05). Values with dissimilar letters are significantly different from each other (*p* < 0.05). Values with the same letter are not significantly different (*p* > 0.05).

XTT assay, which measures the metabolic activity of cells, showed a reduced biofilm metabolic activity by 50% beginning from 12.5 μM concentration while no-metabolic reduction was evidenced at lower concentrations (Figure 3).

These results support the hypothesis that biofilm formation by *C. albicans* was effectively inhibited by the antibiofilm activity of WMR-K, but not by its fungicidal activity, and suggested that unlike conventional fungicides, WMR-K may be less favorable to the development of drug resistance.

However, WMR-K was most potent against *K*. *pneumoniae* compared with *C*. *albicans.* In fact, WMR-K strongly inhibited the growth of *K*. *pneumoniae* (MIC of 10 μM), in addition, it showed a minimum bactericidal concentration (MBC) of 25 μM.

The killing kinetics (Supplementary Figure S1) of WMR-K against *K. pneumoniae* revealed a time-dependent activity and the time required to achieve complete killing of microorganisms after treatment with WMR-K was of 6 h. 

Importantly, WMR-K treatment at 7.5 μM inhibited biofilm formation by >85% reaching an almost total inhibition at 10 μM (Figure 2). Equally, *K. pneumoniae* reduction of biofilm cells metabolic activity was obtained at concentrations between 5 and 10 μM (Figure 3).

These results suggested that the antibiofilm activity of WMR-K was partially caused by antimicrobial activity toward *K*. *pneumoniae.*

Due to the intrinsic recalcitrance of mixed fungal/bacterial biofilms against conventional antibiotic treatment, we were interested in examining the in vitro activity of prevention and treatment of *C. albicans/K. pneumoniae* mature biofilm [11]. In dual-species conditions, WMR-K induced an inhibition of 20% already at lowest concentrations tested showing an excellent preventive activity (Figure 2), while it was necessary to use higher concentrations up to 50 μM to eradicate formed dual-species biofilms approximatively of 80% (Figure 3). 

For the reader’s convenience, the data graphically presented in Figure 2 and Figure 3 are reported in numerical format in Supplementary Tables S1 and S2. 

For a further comprehension of the specific metabolites involved in the complex development process of dual-biofilm formation by *C. albicans/K. pneumoniae*, metabolomic footprinting was used to investigate the dynamic changes of the metabolites and their roles during biofilms inhibition. In particular, we compared metabolites consumption and excretion from the medium between a dual biofilm culture of *C. albicans*/*K. pneumoniae* (control class) and an identical culture treated with 5 μM WMR-K peptide (treated class) after 24 h exposure. A 5 μM WMR-K concentration was carefully selected for the comparative metabolomic study as the most appropriate concentration to maintain the polymicrobial environment in the dual species AMP treated class. In fact, we characterized the polymicrobial biofilm obtained by co-culturing both microorganisms after 24 h treatment with 10 μM WMR-K and observed that *K. pneumoniae* was absent (as could be easily deduced from the above reported MIC for *K. pneumoniae*). However, in presence of 7.5 and 5 μM WMR-K, we observed that *K. pneumoniae* accounted for 1.9 and 9.5%, respectively, of the dual species biofilm.

### 2.2. Metabolomic Analysis

**Figure 4:**
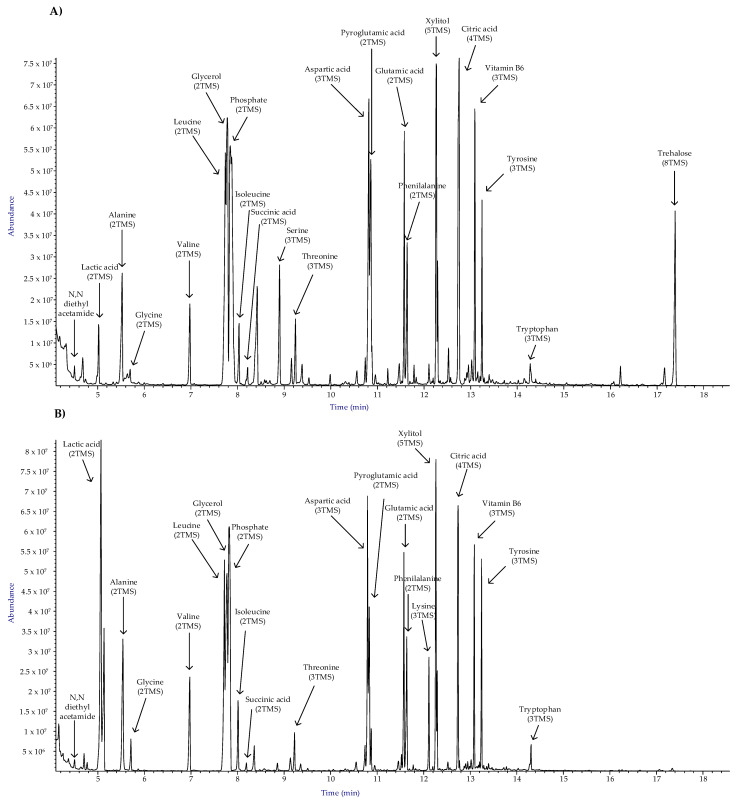
Typical annotated total ion current chromatograms (TICCs) from GC-MS analysis of dual-species biofilm cultures of *C. albicans* and *K. pneumonia* (**A**) in presence and (**B**) absence of the peptide WMR-K (5 µM).

Dual-species biofilm cultures of *C. albicans* and *K. pneumoniae* were grown in the presence and absence of the peptide WMR-K (5 µM) in order to compare their metabolomic profiles after 24 h exposure. The changes in the metabolic profile were determined using the GC-MS based metabolomics tool. Typical reconstructed total ion chromatograms (TICCs) for treated and untreated dual species cultures, respectively, are reported in Figure 4.

In this study, we used an untargeted metabolomic approach, based on AMDIS [39] and SpectConnect [40], which is preferred to compare two different cultural conditions because it allows evaluating the entity of the metabolomic change considering both identified and unidentified compounds avoiding underestimation of differences which actually exist between different conditions.

The final metabolomic dataset, obtained using SpectConnect to track conserved metabolites throughout AMDIS deconvolution results, was composed by 16 observations (8 replicated observations in each class) and 76 conserved variables (metabolites). Technically, in the context of SpectConnect software, a conserved metabolite is one that consistently persists in replicate samples and is said to define a clique. Finally, a 16·76 matrix of autoscaled relative abundances (RA matrix) is created which is submitted to multivariate analysis whose results are presented and discussed below.

**Figure 5:**
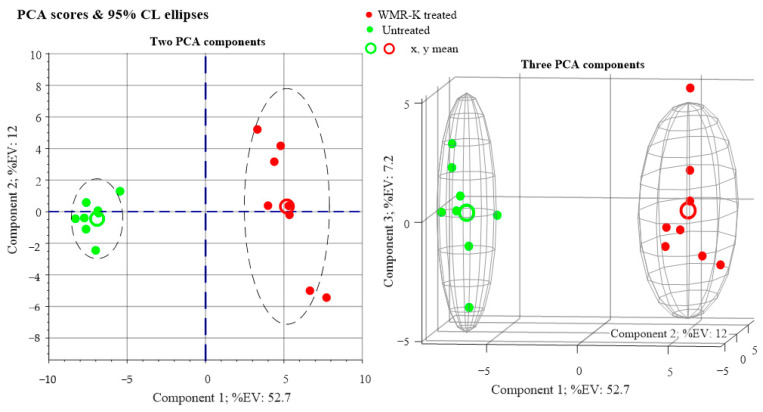
Principal component analysis (PCA) score plots obtained from metabolomic profiles of dual-species biofilms of *C. albicans* and *K. pneumoniae* grown in presence and absence of the peptide WMR-K (5 µM).

Principal component analysis (PCA) was first carried out to detect intrinsic clustering between samples. 2D and 3D PCA score plots in Figure 5 clearly indicate an unsupervised separation between polymicrobial biofilms cultures of *C. albicans* and *K. pneumoniae* in the presence and absence of the peptide (5 µM). 

As can be seen from Figure 5, red bullets (dual species biofilms cultures in medium enriched with a fixed concentration of peptide WMR-K) are well separated on the first principal component axis (PC1) from green bullets, which refer to culture medium not enriched with peptide. Whatever the 1st PCA axis represents, this is convincing evidence that changes in the metabolic profile are caused in the examined cultures by the presence of a small concentration of peptide.

**Figure 6:**
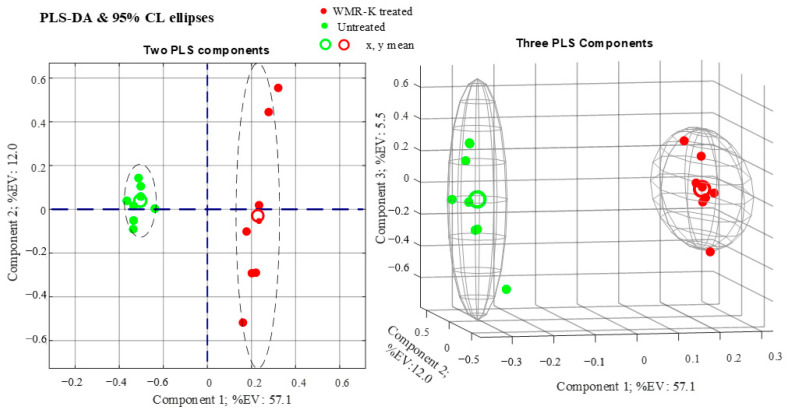
Partial least-squares discriminant analysis (PLS-DA) score plots obtained from metabolomic profiles of dual-species biofilms of *C. albicans* and *K. pneumoniae* in presence and absence of the peptide WMR-K (5 µM).

Subsequently, the conventional supervised method, partial least-squares discriminant analysis (PLS-DA), was performed to maximize the differences between dual-species biofilms obtained in presence and absence of the peptide (Figure 6).

The statistical significance of the PLS model was assessed by the statistics R^2^X, R^2^Y, and Q^2^Y. As can be seen from Figure 6, we obtain R^2^X = 0.7459, which is a noticeably high value taking into account that the data incorporate four biological replicates for each class which are sources of variability in metabolites concentrations. Furthermore, we calculate R^2^Y = 0.9989 and Q^2^Y = 0.9762. The high value of Q^2^Y (evaluated using mean squared errors from 5-fold cross-validation) indicates that the PLS model is a predictive one.

Of the 76 metabolites conserved through biological and technical replicates, a total of 42 components (variables) were identified by comparing their mass spectra with those reported in commercially available libraries and by their Kovats retention index [41]. The 42 identified metabolites are listed in Table 1 with attached relevant information. 

The relative abundances (RA) of each metabolite in the treated and untreated biofilm cultures were submitted to Student *t* test, and the resulting *p* value is reported in Table 1 (which, in fact, is sorted according to ascending *p* values of metabolites in column 2).

A considerable advantage of the PLS-DA multivariate analysis technique is the possibility of calculating the influence that each variable carries on the PLS-DA projection to latent variables (VIP score) which helps in tracking the most important variables for discrimination between classes. In fact, metabolites with VIP score greater than 1 are considered having a statistically significant contribution to the model. Consequently, a VIP score is attached to each metabolite in Table 1. 

**Figure 7:**
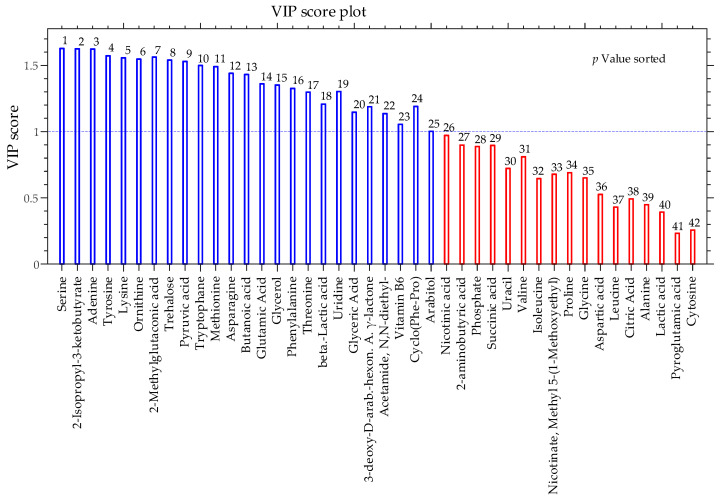
Bar plot of Variable Influence on Projection (VIP) of identified metabolites which contribute to separating dual-species biofilms of *C. albicans* and *K. pneumoniae* in the presence and absence of the peptide WMR-K (5 µM).

Even a superficial inspection of Table 1 and Figure 7 will show that VIP scores and *p* values are strictly correlated (VIP scores decrease as variables become less important for the PLS-DA model while *p* values increase). At a significance level of 0.05, metabolites with *p* value < 0.05 (which translates in VIP scores > 1) have significantly different relative abundances in the two classes.

**Figure 8:**
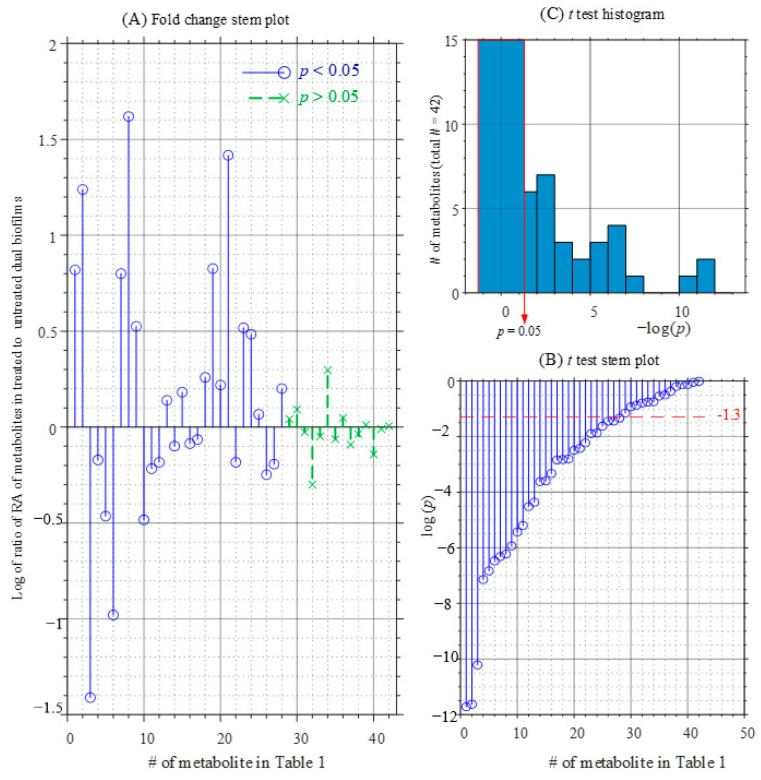
Graphical representations of metabolomic profiles comparison of dual-species biofilms of *C. albicans* and *K. pneumoniae* obtained in the presence and absence of the peptide WMR-K (5 µM): (**A**) fold change stem plot; (**B**) *t*-test *p*-values stem plot; (**C**) *t*-test *p*-values histogram.

From Table 1 and Figure 8, it can be seen that the relative amount of a consistent number of identified metabolites is significantly different in the peptide treated and untreated biofilm. In particular Figure 8A shows that compounds located in the upper panel were positively correlated with the WMR-K-treated class, whereas those located in the opposite are negatively correlated.

It was found that 15 of 29 metabolites, including serine, trehalose, pyruvic acid, glycerol, uridine, glyceric acid, vitamin B6, *cyclo*-(Phe-Pro), and phosphate, were positively correlated to WMR-K-treated class, while the other 14, essentially belonging to the family of amino acids, were negatively correlated. These differentially expressed compounds are mainly related to the primary metabolic pathways. 

Among them, amino acids metabolism seems affected by the presence of the peptide WMR-K in the culture medium. In fact, the vast majority of these metabolites (i.e., tyrosine, lysine, ornithine, tryptophan, methionine, asparagine, glutamic acid, phenylalanine, and threonine) are downregulated in the metabolic profile of the dual species cultures treated with the peptide, with exception of serine. 

Contextually, respect to untreated class, pyruvic acid is up-regulated in the WMR-K treated class while lactate, which lies very low in Table 1 (VIP score = 0.39; *p* value = 0.749) is not significantly different in the two classes. As discussed below, these findings point to an enhanced activity of oxidative metabolism in the WMR-K treated biofilm. 

Furthermore, vitamin B6 and trehalose are up-regulated in the dual-species biofilm culture in presence of the peptide and likely this is connected to their protection role of cells from the stress induced by the AMP.

Lipids metabolism seems also to be affected by the presence of the WMR-K in the culture medium of dual species *C. albicans*/*K. pneumoniae*. In fact, two important intermediates of this pathway were altered in the WMR-K treated class.

The production of some interesting metabolites was stimulated by the peptide. This is the case of *cyclo*-(Phe-Pro), which belongs to the family of diketopiperazine.

## 3. Discussion

To the best of our knowledge, this is the first report about the analysis of the antimicrobial and antibiofilm activities and metabolic features of WMR-K peptide on a dual species biofilm formed by *C. albicans*/*K. pneumoniae*. These pathogens, both leading pathogens in bloodstream and systemic infections representing a major cause of morbidity and mortality in hospitalized patients, are of significant interest because of the escalating development of antimicrobial resistance and the increasing involvement of polymicrobial biofilms in chronic and systemic infections. Moreover, their interaction has been associated with enhanced pathogenic behavior, disease severity, and morbidity [42].

Our study confirms the low antifungal activity but also the good antibacterial activity of WMR-K. On the contrary, the antibiofilm activity showed a maximum biofilm inhibition of 95, 90, and 95% at 10 and 50 μM of WMR-K for *K. pneumoniae*, *C. albicans,* and mixed biofilms, respectively. Results of the XTT assay showed a remaining vital biomass of 20, 30, and 10% only at the highest concentrations tested.

The results of the antimicrobial and antibiofilm activities suggest a promising capacity of low concentrations of WMR-K to inhibit biofilm formation by dual species of *C. albicans/K. pneumoniae* after 24 h exposure. As described above, in order to provide additional evidence for this effect of WMR-K peptide, a study of extracellular metabolites uptake and/or excretion from the culture medium (metabolic footprinting) by the polymicrobial system of *C. albicans* and *K. pneumoniae* in presence and absence of 5 µM of the peptide WMR-K, was performed. 

The cell metabolism is an integrated network of several pathways connected by a complex mechanism of regulation involving several metabolites as intermediates many of which, for a variety of reasons, may not be identified during metabolomic analysis. Therefore, analyses of the metabolomic profiles which rely exclusively on identifiable metabolites are liable to underestimate differences which actually exist between different conditions. For this reason, an untargeted metabolomic approach, based on AMDIS [39] and SpectConnect [40], was used to allow evaluation of the metabolomic change considering both identified and unidentified compounds. A total of 29 identified metabolites were found to be differentially expressed between WMR-K treated and untreated classes, which are mainly involved in the central carbon metabolism (i.e., glycolysis/gluconeogenesis, citrate cycle) and amino acids and lipids metabolism. Hence, diverse metabolomic pathways are affected by the presence of WMR-K in the culture medium.

Serine is the most important identified metabolite which contributes to the discrimination between the two examined classes. In particular, the level of serine in the untreated class is about 1/10 of its level in the WMR-K-treated class. Considering that *K. pneumoniae* is highly sensitive (see Figure 2) to the antibacterial action of WMR-K peptide, the most parsimonious interpretation of this fact is to presume that serine consumption is decreased in the treated class because of an attenuation of *K. pneumoniae* metabolism.

Apart from that, lower levels of serine in dual bacterial/fungal biofilms, with respect to fungal biofilms, are common. For instance, in a previous work performed to elucidate the metabolomic differences between single and polymicrobial biofilm cultures of *P. mirabilis* and *C. albicans* [43], a decrease of about 125 times in the serine level of polymicrobial biofilm cultures, with respect to single species *C. albicans* biofilm, was observed. Finally, it appears that coexistence of *K. pneumoniae* and *C. albicans* increases the uptake of serine in the untreated dual species biofilm with respect to the WMR-K treated biofilm. Obviously, this does not necessarily imply that, in presence of WMR-K peptide, serine uptake from the TSB medium, which contains serine and an assortment of nutrients, is halted. In fact, various amino acids, including ornithine (Fold change = 9.54↓), lysine, tryptophan, tyrosine, methionine, and asparagine (see Table 1), were down-regulated in the WMR-K treated class. The enhanced consumption of these nutrients in the WMR-K treated biofilm might be mainly attributed to *C. albicans,* whose metabolism is manifested in presence of the peptide (due to its antibacterial activity towards *K. pneumoniae*).

Several nutrients, including amino acids, can be metabolized into tricarboxylic acid cycle (TCA) intermediates and enter this cycle. As a consequence, the increased uptake of amino acids may be interpreted as an increased activity of the TCA cycle in WMR-K treated biofilm with respect to the untreated biofilm. 

This interpretation is not in contrast with the observed decreased consumption of pyruvate in WMR-K treated biofilm (Fold change = 3.35↑). In fact, in our view, this may be due to an increased pyruvate production within cells from amino acids which enter the cycle via pyruvate and/or an effect of an increased production of pyruvate from glucose through glycolysis.

Overall, the view of a more active oxidative metabolism in the WMR-K treated biofilm, with respect to untreated biofilm, is well supported.

A possible explanation of this situation might be that the peptide counteracts the formation of biofilm in polymicrobial system maintaining an active oxidative metabolism. This interpretation is consistent with previous studies which stresses the role of amino acids upregulation in the process of biofilm formation and reveal that genes involved in amino acid biosynthesis are upregulated in *C*. *albicans* biofilm compared to planktonic cells [43,44,45].

In aerobically growing organisms, the formation of reactive oxygen species (ROS) is a consequence of an active oxidative metabolism or the presence of external agents [46]. It has been demonstrated that the toxic effects of ROS were opposed by diverse microorganisms accumulating the non-reducing disaccharide trehalose [47,48], the poly-alcohols glycerol and arabitol [49], and vitamin B6 [50,51]. In fact, these compounds, acting as oxygen scavengers, can protect the microorganisms from the oxidative damage, and they have been considered as potential antioxidant.

In this study, we find that trehalose is markedly upregulated in the WMR-K treated biofilm culture with respect to untreated (Fold change ≈ 40↑). 

Upregulation of trehalose in presence of WMR-K peptide is such a prominent phenomenon that is visible to the bare eye by comparing the well-developed and outstanding peak of trehalose in the chromatogram in Figure 4A with the corresponding peak in the chromatogram of Figure 4B.

A previous work on *C. albicans* pointed to a specific role of trehalose in cellular protection against amphotericin B, a well-known antifungal agent, which is thought to act by inducing oxidative stress in *C. albicans* cells. Accumulation of trehalose in early and intermediate phase *C. albicans* biofilms, treated with amphotericin B, was interpreted as one important factor contributing to drug resistance [52]. Therefore, the ~40-fold increase of trehalose in the peptide treated group might be a manifestation of *C. albicans* metabolism. 

It is likely that trehalose upregulation in WMR-K treated *K. pneumoniae/C. albicans* biofilms represents an answer of the fungus to reduce antifungal action of the AMP. Up-regulation of vitamin B6 in the treated biofilm (Fold change = 3.6↑) could be an additional factor contributing to the survival of *C. albicans* cells under the stress induced by the peptide. Finally, up-regulation of trehalose and vitamin B6 are a viable explanation for the moderate antifungal action of WMR-K peptide reported above.

Lipids play a key role in the regulation of cell metabolism and are essential energy storage molecules. It was reported that antimicrobial agents modify the levels of the intermediates involved in the lipids metabolism in cultures of *C. albicans* [53]. Our results seem to support this view because the presence of the WMR-K in the culture medium alters the levels of glyceric acid, butanoic acid, and glycerol, directly involved in this biosynthetic pathway.

Interestingly, the cyclic dipeptide *cyclo*-(Phe-Pro) was conserved only in WMR-K treated class. This compound belongs to the family of diketopiperazines and was identified as product of the secondary metabolism of several microorganisms [54,55] regulating the expression of genes involved in the pathogenicity [55]. This evidence can be explained with a possible effect of the peptide WMR-K on the secondary metabolism, which might be the object of further studies by the application of target metabolomics analysis.

## 4. Materials and Methods

### 4.1. Microbial Strains and Cultural Conditions

*Candida albicans* ATCC 90028 and *Klebsiella pneumoniae* ATCC 10031 (Manassas, VA, USA) were used in this study. *C. albicans* was grown in Tryptone Soya Broth (TSB) supplemented with 0.1% glucose, at 37 °C, for 16–18 h, and *K. pneumoniae* was grown in Tryptone Soya Broth (TSB) (Merck, Darmstadt, Germany) without glucose at 37 °C, for 16–18 h, and they were respectively maintained in Sabouraud dextrose agar and Triptone Soy Agar (TSA) (Merck, Darmstadt, Germany) containing glycerol at −80 °C. Overnight cultures of *C. albicans* and *K. pneumoniae* were washed twice using sterile phosphate buffered saline (PBS) and standardized to 10^6^ cells·mL^−1^ for next experiments.

### 4.2. Peptide Synthesis 

Peptide WMR-K (NH2-WGIRRILKYGKRSK-CONH2) was prepared through the Fmoc-based solid-phase method, using a rink amide MBHA (0.57 mmol·g^−1^) resin. Several cycles of coupling (2 equivalents of amino acid +2 equivalents of 1-hydroxybenzotriazole (HOBT)/2-(1H-Benzotriazole-1-yl)-1,1,3,3-tetramethyluronium hexafluorophosphate (HBTU) (0.45 M in DMF) for 30 min (2×)) and deprotection (30% piperidine in dimethylformamide –DMF-, 5 min (2)) were performed.

Side chain deprotection and cleavage from the resin was performed by treatment with an acid solution composed by trifluoroacetic acid (95% *v/v*): H_2_O (2.5 *v/v*): Triisopropylsilane (2.5 *v/v*) for 6h at room temperature. Following deprotection, the crude peptide was precipitated in cold ethylic ether and purified by preparative RP-HPLC (Shimadzu Corporation, Kyoto, Japan). Peptide was obtained with good yields (50%), and its identity was confirmed using LTQ-XL linear ion trap mass spectrometry (Thermo Scientific, Waltham, MA, USA).

### 4.3. Minimum Inhibitory Concentration and MBC/MFC

The minimum inhibitory concentration (MIC) of WMR-K against *C. albicans* and *K. pneumoniae* was determined using a microbroth dilution assay as per CLSI guidelines [56] with some modifications. Briefly, 100 μL of nutrient broth with or without glucose containing each strain to a final concentration of 1 × 10^6^ colony-forming units (CFU)·mL^−1^ was introduced into each well of 96- well microplate and different concentrations of WMR-K between 5 and 50 µM and 5 to 200 µM were introduced into the wells and incubated for 24 h at 37 °C. The antimicrobial activity of peptide was evaluated by measuring the absorbance of the cells at 590 nm wavelength using a microplate reader (Synergy^TM^ H4; BioTek Instruments, Inc., Winooski, VT, USA). The MIC endpoint is the lowest concentration of WMR-K capable of inhibiting the growth of each microbial pathogen. Fungal solution and liquid medium were used as positive control group, while liquid medium was used as negative control group. Amphotericin B and rifamycin (Sigma-Aldrich, St. Luis, MO, USA) were selected as positive control drugs. 

In addition, 10 μL from all wells which showed no overnight growth were plated on Sabouraud dextrose/TSA agar plates and incubated at 37 °C for 24 h to determine the minimum fungicidal concentration (MFC) and the minimum bactericidal concentration (MBC), defined as the lowest concentration of WMR-K required to kill 99.9% of cells when compared with initial inoculum by colony forming unit (CFU) counting. The effect was considered fungicidal/bactericidal when MFC/MBC was ≤4× MIC; otherwise, the effect was considered fungistatic or bacteriostatic [57]. All experiments were performed in triplicate.

### 4.4. Time to Kill Assays

Time-kill kinetics of WMR-K of *C. albicans* and *K. pneumoniae* was carried out at different time points. Briefly, different concentrations of WMR-K for bacteria (5, 7.5, 10, and 12.5 µM) and fungi (25, 50, 100, and 200 µM) were prepared. An inoculum size of 1·10^6^ CFU·mL^−1^ was added and incubated at 37 °C until 24 h. Aliquots of cell suspensions were taken at time intervals of 6, 12 and 24 h. CFU was determined after incubation for 24/48 h at 37 °C. The procedure was performed in triplicate (three independent experiments).

### 4.5. Biofilm Formation and Quantification

Biofilms were developed according to the modified microtiter plate test proposed by Stepanovic et al. [38], with some modifications.

Cell suspensions of *K. pneumoniae* or *C. albicans* were adjusted to 1·10^6^ CFU·mL^−1^ with TSB. For monomicrobial biofilms, 100 μL of bacterial or fungal culture was added in 96-well microplate. For polymicrobial biofilms, both microorganism suspensions were mixed 1:1 and added to the 96-well microplate. The plates were incubated at 37 °C for 24 h under static conditions and biofilm formation was visualized by staining with 0.1% crystal violet. Biofilm formation was quantified as an optical density (OD570) after solubilization with 30% acetic acid. 

### 4.6. Quantification of Mixed Biofilm by Colony Forming Units (CFUs)

The quantification of the number of viable cells in the polymicrobial biofilms was based on the methodologies described by Vilela et al. with some modification [58]. Briefly, biofilms, both 24 and 48 h, were gently washed twice with sterile PBS, homogenized and scraped up adding 200 μL of sterile PBS.

Cells were serially diluted, aliquots were inoculated into Petri dishes containing TSA (with amphotericin B, 1 μg·mL^−1^) or Rose Bengal (with chloramphenicol, 1 μg·mL^−1^) agar plates and then incubated at 37 °C for 24/48 h before counting. 

### 4.7. Inhibition and Eradication of WMR-K on Mono- and Polymicrobial Biofilms

For biofilm inhibition assay, the microbes alone or together were incubated with different sub-MIC concentrations of WMR-K (5, 7.5, and 10 μM for *K pneumoniae* and 5, 7.5, 10, 12.5, 25, and 50 μM for *C. albicans* and polymicrobial culture) for 24 h. Biofilms total biomass was quantified as previously described by using crystal violet [38]. Hence, media with planktonic cells were discarded; wells were washed three times with PBS and air dried for 30 min, and 150 μL of 0.1% crystal violet solution was added to each well and incubated at room temperature for 20 min. After washing with distilled water, 150 μL of 30% acetic acid was added to each well, and absorbance was measured at 570 nm using a microplate reader. The mean absorbance values of each sample were calculated and compared with the mean value of the control (microorganisms without WMR-K). 

In order to test the effect against established biofilm, fresh growth medium containing WMR-K at the same concentrations was added in the preformed 24 h old-biofilm and incubated for another 24 h. Biofilms vital biomass were quantified using the tetrazolium 2,3-bis(2-methoxy-4-nitro-5 sulfophenyl)-5-[(phenylamine) carbonyl]- 2H-hydroxide reduction assay (XTT) (Sigma-Aldrich, St. Luis, MO, USA) according to the manufacturer’s instructions. Plates were assessed by colorimetric changes read at 492 nm. Statistical analyses were performed using GraphPad Prism Software (version 8.02 for Windows, GraphPad Software, La Jolla, CA, USA). All assays were performed in triplicates, and all the results were reported as a mean ± standard deviation (SD). ANOVA test was used to compare the differences within and between treatments after normality and homoscedasticity verification (Shapiro–Wilk test and F-test) followed by Tukey’s test for multiple comparisons. (*p* < 0.05). 

### 4.8. Metabolomic Analysis

#### 4.8.1. Sample Preparation

For metabolomic profiling, the dual-species biofilm cultures of *C. albicans* and *K. pneumoniae* were grown in microtiter plates in the presence and absence of the peptide WMR-K (5 µM) for 24 h, which constitutes two classes of samples. After the incubation period, the culture supernatants were transferred into Eppendorf tube and centrifuged. The samples were completely dried with a stream of nitrogen, and the residues were treated with *N*,*O*-bis(trimethylsilyl)-trifluoroacetamide (BSTFA) (Fluka, Buchs, Switzerland) as previously described [59]. Each class comprises four biological replicates. Furthermore, GC-MS analysis of each biological replicate was replicated two times, giving in total 16 samples for metabolomic profiling.

#### 4.8.2. GC-MS Analysis

Trimethylsilyl derivatives were analyzed by an Agilent 6850 GC (Milan, Italy), equipped with an HP-5MS capillary column (5% phenyl methyl poly siloxane stationary phase), coupled to an Agilent 5973 Inert MS detector operated in the full scan mode (*m/z* 29–550) at a frequency of 3.9 Hz and with the EI ion source and quadrupole mass filter temperatures kept, respectively, at 200 and 250 °C. Helium was used as carrier gas at a flow rate of 1 mL·min^−1^. The injector temperature was 250 °C and the temperature ramp raised the column temperature from 70 to 280 °C: 70 °C for 1 min; 10 °C·min^−1^ until reaching 170 °C; and 30 °C·min^−1^ until reaching 280 °C. Then, it was held at 280 °C for 5 min. The solvent delay was 4 min.

#### 4.8.3. Data Processing and Statistical Analysis

Raw data from GC-MS analysis were deconvoluted using the NIST program AMDIS (Automated Mass Spectral Deconvolution and Identification System) [39], and then, the conserved metabolites peaks across each biological and technical replicate were listed and tracked using SpectConnect [40]. The metabolites relative abundances (RA) were auto-scaled, and the created dataset was then submitted to multivariate statistical analyses, such as principal component analysis (PCA) and partial least squares discriminant analysis (PLS-DA), which were performed with MATLAB (Mathworks, Natick, MA, USA) [60] and a homemade .m script. Variables significantly contributing to the clustering and discrimination of samples were identified according to variable influence on projection (VIP) values generated by PLS-DA processing. Meanwhile, differentially expressed metabolites were detected by Student’s *t*-test with the *p* value of less than 0.05.

Metabolites were identified by comparing their EI mass spectra at 70 eV with spectra of known substances present in the NIST 14 mass spectral library [61] and the Golm metabolome database [62,63]. Furthermore, the identification was supported by Kovats retention index (RI) calculated for each analyte by the Kovats equation, using the standard *n*-alkane mixture in the range C7-C40 (Sigma-Aldrich, Saint Louis, MO, USA) [41].

## Figures and Tables

**Table 1:** Identified metabolites from metabolomic analysis on two classes of samples: dual biofilm cultures of *C. albicans* and *K. pneumoniae* in the presence (class 1) and absence (class 2) of the peptide WMR-K (5 µM). Metabolites (variables) are sorted in ascending order of *t* test *p*-value. Arrows indicate the direction (trend) of fold change comparing the two classes: (↑), upregulated in presence of the peptide WMR-K; (↓), downregulated in presence of the peptide WMR-K; (=) no statistically significant change. RI represents Kovats retention index and TMS is the trimethylsilyl function, (CH_3_)_3_Si-.

	Metabolite	VIP Score	*t* Test ‘*p*-Value’	Trend	Fold Change	Functional Category
1	Serine, 3TMS (RI: 1375)	1.627	1.94 × 10^−12^	↑	8.54	Amino acid metab.
2	2-Isopropyl-3-ketobutyrate, 2TMS (RI: 1463)	1.625	2.40 × 10^−12^	↑	17.34	−
3	Adenine, 2TMS (RI: 1890)	1.623	6.20 × 10^−11^	↓	25.75	Nucleotide and energy metab.
4	Tyrosine, 3TMS (RI: 1962)	1.572	7.26 × 10^−8^	↓	1.48	Amino acid metab.
5	Lysine, 3TMS (RI: 1722)	1.557	1.48 × 10^−7^	↓	2.91	Amino acid metab.
6	Ornithine, 3TMS (RI: 1632)	1.547	3.40 × 10^−7^	↓	9.54	Amino acid metab.
7	2-Methylglutaconic acid, 3TMS (RI: 1149)	1.562	4.88 × 10^−7^	↑	6.32	−
8	Trehalose, 8TMS (RI: 2810)	1.540	5.99 × 10^−7^	↑	41.65	Stress response
9	Pyruvic acid, 2TMS (RI: 1108)	1.530	1.18 × 10^−6^	↑	3.35	Glycolysis/ gluconeogenesis
10	Tryptophan, 3TMS (RI: 2253)	1.499	3.70 × 10^−6^	↓	3.04	Amino acid metab.
11	Methionine, 2TMS (RI: 1536)	1.491	6.41 × 10^−6^	↓	1.65	Amino acid metab.
12	Asparagine [-H2O], 2TMS (RI: 15616)	1.440	3.06 × 10^−5^	↓	1.53	Amino acid metab.
13	Butanoic acid, 3TMS (RI: 1425)	1.431	4.40 × 10^−5^	↑	1.38	Lipid metab.-
14	Glutamic acid, 3TMS (RI: 1638)	1.361	2.42 × 10^−4^	↓	1.26	Amino acid metab.
15	Glycerol, 3TMS (RI: 1290)	1.352	2.55 × 10^−4^	↑	1.52	Stress response, lipid metab.
16	Phenylalanine, 2TMS (RI: 1647)	1.326	4.74 × 10^−4^	↓	1.22	Amino acid metab.
17	Threonine, 3TMS (RI: 1400)	1.298	1.46 × 10^−3^	↓	1.16	Amino acid metab.
18	*Beta*-lactic acid, 2TMS (RI: 1156)	1.208	1.50 × 10^−3^	↑	1.82	Stress response
19	Uridine, 3TMS (RI: 2594)	1.302	1.58 × 10^−3^	↑	6.71	Nucleotide metab.
20	Glyceric acid, 3TMS (RI: 1346)	1.146	3.22 × 10^−3^	↑	1.66	Lipid metab.
21	3-Deoxy-D-arabino-hexonic acid γ-lactone, 3TMS (RI: 1797)	1.188	3.74 × 10^−3^	↑	26.13	-
22	Acetamide, N,N-diethyl- (RI: 1045)	1.136	5.98 × 10^−3^	↓	1.53	-
23	Vitamin B6, 3TMS (RI: 1924)	1.055	1.29 × 10^−2^	↑	3.29	Stress response
24	*Cyclo-*(Phe-Pro) (RI: 2434)	1.190	1.34 × 10^−2^	↑	3.05	Secondary metab.
25	Arabitol, 5TMS (RI: 1750)	1.002	2.39 × 10^−2^	↑	1.17	Glycolysis/ gluconeogenesis
26	Nicotinic acid, TMS (RI: 1304)	0.971	3.60 × 10^−2^	↓	1.77	Nicotinate metab
27	2-Aminobutyric acid, 2TMS (RI: 1149)	0.898	3.64 × 10^−2^	↓	1.56	Amino acid metab.
28	Phosphate, 3TMS (RI: 1297)	0.888	4.64 × 10^−2^	↑	1.59	Energy metab.
29	Succinic acid, 2TMS (RI: 1322)	0.896	6.97 × 10^−2^	↑=	1.10	Citrate cycle
30	Uracil, 2TMS (RI: 1351)	0.722	1.18 × 10^−1^	↑=	1.23	Nucleotide metab.
31	Valine, 2TMS (RI: 1230)	0.810	1.35 × 10^−1^	↓=	1.06	Amino acid metab.
32	Isoleucine, 2TMS (RI: 1307)	0.645	1.62 × 10^−1^	↓=	1.99	Amino acid metab.
33	Nicotinate, methyl 5-(1-Methoxyethyl) (RI: 1354)	0.678	1.75 × 10^−1^	↓=	1.12	Nicotinate metab.
34	Proline, 2TMS (RI: 1314)	0.690	1.80 × 10^−1^	↑=	1.98	Amino acid metab.
35	Glycine, 2TMS (RI: 1136)	0.650	2.87 × 10^−1^	↓=	1.15	Amino acid metab.
36	Aspartic acid, 3TMS (RI: 1540)	0.526	3.16 × 10^−1^	↑=	1.12	Amino acid metab.
37	Leucine, 2TMS (RI: 1286)	0.430	4.23 × 10^−1^	↓=	1.24	Amino acid metab.
38	Citric acid, 4TMS (RI: 1844)	0.492	6.38 × 10^−1^	↓=	1.14	Amino acid metab.
39	Alanine, 2TMS (RI: 1124)	0.448	7.34 × 10^−1^	↑=	1.03	Amino acid metab.
40	Lactic acid, 2TMS (RI: 1083)	0.392	7.49 × 10^−1^	↓=	1.39	Stress response
41	Pyroglutamic acid, 2TMS (RI: 1546)	0.232	8.96 × 10^−1^	↓=	1.02	Amino acid and glutathione metab.
42	Cytosine, 2TMS (RI: 1546)	0.256	9.72 × 10^−1^	↑=	1.01	Nucleotide metab.

## Data Availability

Not applicable.

## Supplementary Materials

The following are available online at https://www.mdpi.com/2076-0817/10/2/214/s1. Figure S1: Time-kill kinetics of WMR-K against *C. albicans* and *K. pneumoniae*. Table S1: Inhibition effects of WMR-K at different concentrations on mono and poly-microbial biofilms of *K. pneumoniae* and *C. albicans*. Table S2: Eradication effects of WMR-K at different concentrations on mono and poly-microbial biofilms of *K. pneumoniae* and *C. albicans.*
