# Supplementary material for: Impact of the Peptide WMR-K on Dual-Species Biofilm Candida albicans/Klebsiella pneumoniae and on the Untargeted Metabolomic Profile
Source: Pathogens. 2021 Feb 16;10(2):214. doi: 10.3390/pathogens10020214 (PMC7920046; doi:10.3390/pathogens10020214)
Supplement: Supplementary file 1 [file pathogens-10-00214-s001.pdf]

Impact of the peptide WMR-K on dual-species biofilm *Candida albicans*/*Klebsiella pneumoniae* and on the untargeted metabolomic profile

Emilia Galdiero [1], Maria Michela Salvatore [2*], Angela Maione [1], Federica Carraturo [1], Stefania Galdiero [3], Annarita Falanga [4], Anna Andolfi [2,5], Francesco Salvatore [2] and Marco Guida [1]

[1] Department of Biology, University of Naples 'Federico II', via Cinthia, 80126, Naples, Italy; angela.maione@unina.it (A.M.); federica.carraturo@unina.it (F.C.); marco.guida@unina.it (M.G.); egaldier@unina.it (E.G.)

[2] Department of Chemical Sciences, University of Naples 'Federico II', via Cinthia, 80126 Naples, Italy; andolfi@unina.it (A.A.); frsalvat@unina.it (F.S.)

[3] Department of Pharmacy, School of Medicine, University of Naples 'Federico II', Via Domenico Montesano 49, 80131 Naples, Italy; sgaldier@unina.it (S.G.)

[4] Department of Agricultural Science, University of Naples 'Federico II', Via dell' Università 100, 80055, Portici, Naples, Italy; annarita.falanga@unina.it (A.F.)

[5] BAT Center – Interuniversity Center for Studies on Bioinspired Agro-Environmental Technology, University of Naples 'Federico II', Portici (NA), 80055, Italy

* Correspondence: mariamichela.salvatore@unina.it (M.M.S.)

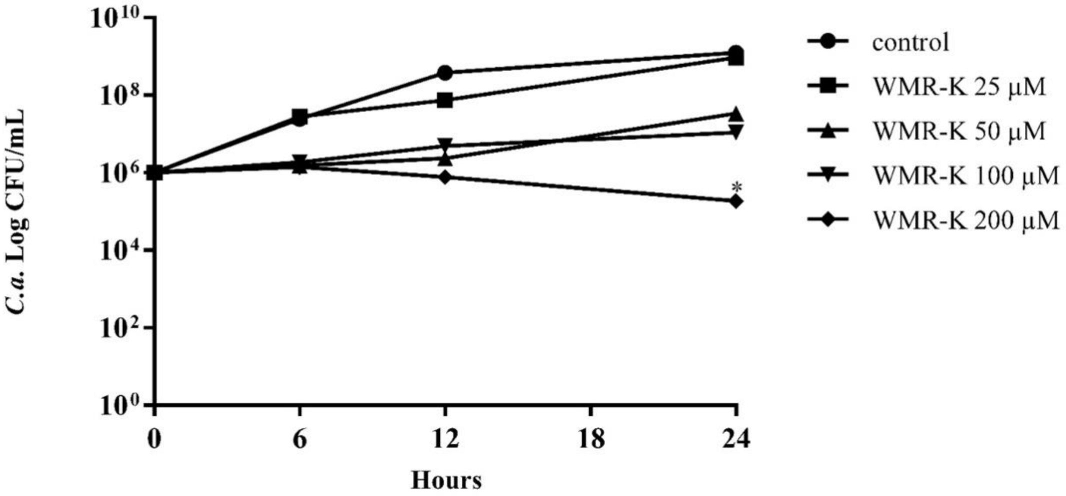

A

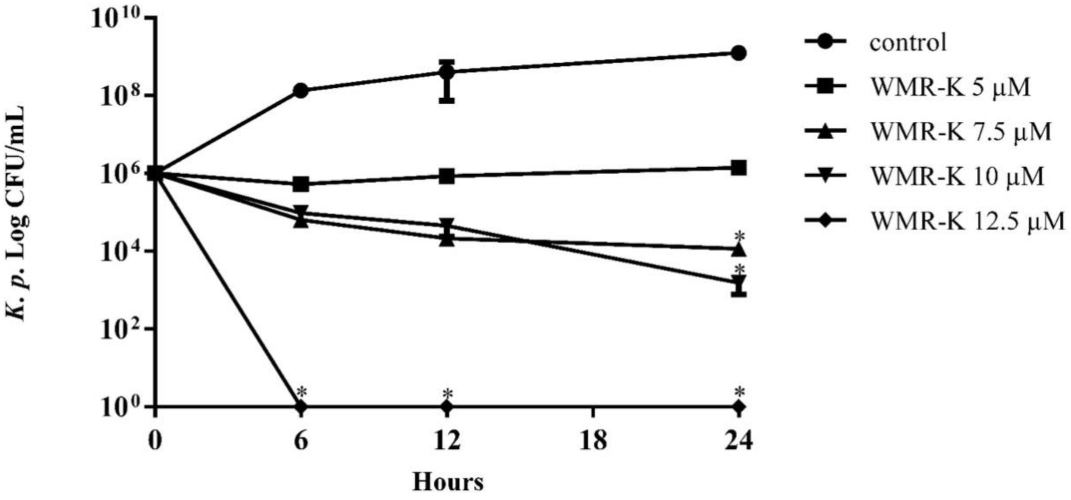

B

Figure S1. Time-kill kinetics of WMR-K against *C. albicans* and *K. pneumoniae.* (A) Growth curves generated using *C. albicans* cells treated with WMR-K. (B) Growth curves generated using *K. pneumoniae* cells treated WMR-K. (n=3 ± SD); * $p < 0.05$ (two-way ANOVA followed by Tukey's post hoc test).

Table S1. Inhibition effects of WMR-K at different concentrations on mono and poly-microbial biofilms of *K. pneumoniae* and *C. albicans*. *n* = number of replicates; SD = standard deviation.

Inhibition effects of WMR-K				
**Culture**	**WMR-K [μM]**	***n***	**Mean (%)**	**SD**
K. pneumoniae	5.0	3	30.00	9.89
	7.5	3	73.50	3.53
	10	3	93.76	3.90
C. albicans	5.0	3	12.01	1.39
	7.5	3	38.50	3.53
	10.0	3	55.5	4.94
	12.5	3	73.00	5.65
	25.0	3	80.00	1.41
	50.0	3	87.01	1.39
Dual species *C. albicans/K. pneumoniae*	5.0	3	18.5	4.94
	7.5	3	31.00	2.82
	10.0	3	63.92	2.93
	12.5	3	78.00	1.41
	25.0	3	87.50	2.12
	50.0	3	89.50	3.53

Table S2. Eradication effects of WMR-K at different concentrations on mono and poly-microbial biofilms of *K. pneumoniae* and *C. albicans*. *n* = number of replicates; SD = standard deviation.

Eradication				
**Culture**	**WMR-K [μM]**	***n***	**Mean**	**SD**
K. pneumoniae	5.0	3	42.00	8.48
	7.5	3	54.50	4.94
	10	3	81.59	7.90
C. albicans	5.0	3	0.00	0.00
	7.5	3	1.00	1.41
	10.0	3	20.91	4.11
	12.5	3	32.50	4.94
	25.0	3	48.00	5.65
	50.0	3	67.64	3.73
Dual species *C. albicans/K. pneumoniae*	5.0	3	0.00	0.00
	7.5	3	14.50	3.53
	10.0	3	39.72	2.43
	12.5	3	58.00	1.41
	25.0	3	70.82	6.82
	50.0	3	82.50	4.94
